# Experiences and needs concerning health related information for newly arrived refugees in Sweden

**DOI:** 10.1186/s12889-020-09163-w

**Published:** 2020-07-01

**Authors:** L. Mårtensson, P. Lytsy, R. Westerling, J. Wångdahl

**Affiliations:** 1 Institute of Neuroscience and Physiology, Sahlgrenska Academy, University of Gothenburg, PO Box 455, 405 30 Göteborg, Sweden; 2 Department of Public Health and Caring Sciences, Uppsala University, Science Park, Box 564, 751 22 Uppsala, Sweden; 3 Division of Insurance Medicine, Department of Clinical Neuroscience, The Karolinska Institute, Stockholm, 17177 Sweden

**Keywords:** Asylum seekers, Health information, Health literacy, Migration, Minority groups

## Abstract

**Background:**

Owing to communication challenges and a lack of knowledge about the health care system, refugees may be at risk of having limited health literacy, meaning that they will have problems in achieving, understanding, appraising and using health information or navigating in the health care system. The aim of this study was to explore experiences and needs concerning health related information for newly arrived refugees in Sweden.

**Methods:**

A qualitative design with a focus group methodology was used. The qualitative content analysis was based on seven focus group discussions, including 28 Arabic and Somali speaking refugees.

**Results:**

Four categories emerged. ‘Concrete instructions and explanations’ includes appreciation of knowledge about how to act when facing health problems. ‘Contextual knowledge’ comprises experienced needs of information about the health care system, about specific health risks and about rights in health issues. ‘A variation of sources’ describes suggestions as to where and how information should be given. ‘Enabling communication’ includes the wish for more awareness among professionals from a language and cultural point of view.

**Conclusion:**

Concrete instructions and explanations are experienced as valuable and applicable. Additional information about health issues and the health care system is needed. Information concerning health should be spread by a variety of sources. Health literate health organizations are needed to meet the health challenges of refugees, including professionals that emphasize health literacy.

## Background

Health literacy concerns both the personal skills and social resources needed to achieve, understand, appraise and use health related information and services as a base for well-founded decision-making and to navigate in the health care system [1]. Health literacy can affect health behavior and the use of health care, which in turn can affect health costs and health outcomes. Furthermore, health literacy can directly or indirectly influence participation and empowerment, and thus affect the equity and sustainability of change in public health [2].

Health information in today’s society is generated and received from various sources, such as mass media, social medias, advertisements, the Internet, the educational system and by health care providers [3]. The quality of the sources varies, and it may be difficult for an individual to know which source may be relied upon and which not. Furthermore, people may have limited language skills which together with poor quality of translations and interpreters further affects their apprehension [4–7]. Health information and information about health has also become more advanced, technical, and complex [8] due to the specialization and diversity of health care and the many professionals involved.

Migrants, also in Sweden, must never be regarded as a homogeneous group as to their health needs and health care utilization. Migrants that come from non-European countries are, for example, known to be worse off when reporting self-assessed health, with this being even more pronounced in those with a refugee background, increasing their vulnerability for psychiatric disease [9]. According to data from 2014, health care utilization was somewhat lower than in the rest of the population, although with relatively much higher utilization of child and adolescent psychiatry [10]. In terms of health literacy, migrants, specifically refugees are a vulnerable group [11–14]. This vulnerability may have its explanation in language and communication problems that make it problematic to understand health information. However, it may also be explained in terms of unawareness about how to access reliable health information and a lack of knowledge about the health care system [15–20]. Studies confirm that refugees in Sweden describe a lack of information about the health care system and their rights to health care [21], as well as a lack of knowledge about where to go for counselling in health matters [22]. The influx of refugees has increased world-wide in recent years [4]; in 2015, there were about 24.6 million refugees, i.e. persons who have fled from and/or cannot return to their home country due to a well-founded fear of persecution, including war or civil conflict [4]. This increase of refugees indicates that the issue of health literacy challenges among those is a burning question.

One source of valuable health information for newly arrived refugees is the Health Examination for Asylum seekers (HEA), an examination provided in many countries. Its central purpose is to identify poor health in order to secure the well-being of the asylum seekers and to guarantee the safety of the population in the host country [5]. However, it also serves as an opportunity to inform asylum seekers about the health system in the new country to provide them with better access to health care [6–8]. The content of an HEA and whether it is mandatory or voluntary varies between countries [5]. Another source of information on health and health systems in the new environment is courses in civic orientation arranged for newly arrived migrants [23]. Both HEA and courses in civic orientation with their health information may be regarded as a societal or situational factor that may have an impact on or be dependent on a person’s health literacy [2].

This study is part of a project that examines different aspects of health literacy among newly arrived refugees [24]. Data from a quantitative study in this project show that a considerable proportion of Arabic-, Dari- or Somali-speaking refugees had limited health literacy and bad experiences of the communication in and the usefulness of information given in the HEA [25]. The aim of this study was to explore experiences and needs concerning health related information in general among refugees in Sweden.

## Methods

### Study design and setting

An explorative, qualitative design was used [26]. This design is used to gain insight into phenomena to which there are few earlier studies to refer. Focus groups were employed to generate qualitative data [27, 28]. This data collection technique is used to examine people’s experiences [28, 29]. It is often used in cross cultural research and in work with ethnic minorities, as it is particularly sensitive to cultural variations [28]. The informal design of focus groups stimulates new ideas and insight on the part of participants and can provide information that the researcher may not have considered [30]. Focus groups highlight the collective view, not the individual view [29], even though individual experiences become clear in the discussions [28].

The research group consisted of four Swedish-born researchers without a migrant background, two women and two men. At the time of the study, the group included a senior professor, an associate professor, a senior researcher and a Ph.D. student, with experience of both research and practice in the fields of health promotion, migration, health literacy and rehabilitation. Regarding the method chosen in this study, one of the researchers had extensive experience of qualitative research. Two of the researchers (first and last authors) have extensive knowledge and experience of research in the area of health literacy.

The data collection phase was carried out in four Swedish cities and was based on purposive selection; the participants were recruited from language courses for migrants and/or civic orientation courses. An application for ethical approval was sent to the regional Ethical Committee of Clinical Investigation in Uppsala. However, a committee judgment was not deemed necessary or applicable according to Swedish law (Dnr: 2013:446).

### Recruitment process

Inclusion criteria for participation in the focus group discussions were: speaking Arabic or Somali fluently, having received a permanent resident permit as a seeker of asylum and having participated in an HEA in Sweden in the last 3 years. The language groups were chosen on the basis oftheir predominance among refugees in Sweden at the time of the study [10].

Four schools offering courses in Swedish for immigrants (SFI) and three centers offering civic orientation [23] were contacted and verbally informed about the study, and all decided to participate. Information was then mediated to participants in the courses together with written detailed information and informed consent forms in Arabic and Somali. In addition, prospective participants and cultural mediators in the civic orientation courses were asked to invite other people that they knew fulfilled the inclusion criterion. The written information included a description of the aim of the study, the aspects of voluntary participation, the possibility to withdraw at any time without explanation, and the confidentiality of the handling and presentation of data. Those who showed an interest in participating were asked for their informed consent and information about country of birth, resident permit, gender, age and education level. They were then contacted and given practical information about the time and procedure of the focus group discussion. On site, before the focus group discussion, the participants were orally informed about the focus group design and the moderator’s role in the discussions. No compensation was offered for participating in the study.

### Participants

In total, 28 persons consented to participate in the study. The number of people who declined to participate was not recorded because the teachers gave information about the study to large groups. The participants were divided into seven focus groups with three to five participants in each group; four had Arabic-speaking participants and three had Somali-speaking participants. The discussions were carried out in six different places in the four cities. Most participants had received a permanent resident permit in the previous 2 years. Detailed characteristics of the participants are presented in Table 1.

**Table 1:** Description of the participants in the focus group discussions (*n* = 28)

	Group 1 *n*=	Group 2 *n*=	Group 3 *n*=	Group 4 *n*=	Group 5 *n*=	Group 6 *n*=	Group 7 *n*=
**Gender**							
Female	–	5	3	–	4	5	–
Male	4	–	–	3	–	–	4
**Age**							
Mean (range)	44 (41–54)	40 (37–42)	missing*	29 (24–37)	36 (27–55)	34 (28–49)	51 (31–67)
**Education (years)**							
No education/illiterate	–	–		–	–	1	2
1-6 years	–	–		–	1	2	2
7-9 years	–	–	missing**	1	1	2	–
10–12 years	1	5		–	1	–	–
> 12 years	3	–		2	1	–	–
**Country of birth**							
Palestine	–	2	–	–	–	–	–
Jordania	–	1	–	–	–	–	–
Somalia	–	–	–	–	4	5	4
Syria	4	2	3	3	–	–	–
**Native language**							
Arabic	4	–	3	3	–	–	–
Somali	–	5	–	–	4	5	4

* Three participants were between18 and 65; exact ages are missing. ** Three participants had < 10 years of education; exact level is missing

Both homogeneity and heterogeneity in the composition of the focus groups are recommended to stimulate the discussions [29, 30]. Homogeneity was ensured by the participants’ common experience of being a refugee and by having taken part in an HEA or a civic orientation course. This homogeneity was used to stimulate the discussions. As heterogeneity is an important factor for covering the diversity within the target group [28], participants with differences concerning gender, level of education and geographical origin were mixed in the groups.

### Data collection

The focus group discussions were carried out group-wise in a quiet and calm environment. They were moderated by one Arabic speaking and two Somali-speaking female moderators, who all had appropriate academic degrees and cultural competence [31]. All except one of the moderators had previous experience of moderating focus groups in research projects in public health. Prior to moderating the discussions, they participated in an educational session including focus group methodology, ethics and the purpose and background of the study. Neither moderators nor researchers had any kind of relationship with the participants prior to the study.

A guide was developed to support the moderators in carrying out the discussions [32]. This guide was developed by the research group specifically for the study. It included discussion questions about the participants’ own experiences of the usefulness, accessibility, clarity and applicability of health information received after their arrival in Sweden. The guide also included discussion questions about the experienced needs concerning the content and distribution of health information and about ways to improve the exchange of health information. A detailed description of the question discussed is presented in the Discussion guide (Additional file 1).

The guide was translated to Arabic. When it had been used in the first focus group, it was considered appropriate and was thus also translated into Somali. Before the beginning of the discussions, the participants were informed that neither the moderator nor the researcher worked in Swedish health care or at the Swedish Migration Agency, to minimize the risk of an influence of a state of dependence in the discussions.

One of the researchers (the last author) took part in the focus groups as an observer during the sessions. She did not understand Arabic or Somali but acted as support for the moderator. As an example, she reminded the moderator about the intention of equal contribution and engagement among the participants in the discussions when some participants became too dominant. She also gave support by answering supplementary questions from the moderators to help them keep their focus based on the aim.

After each discussion, the respective moderator and this researcher summed up the content of what was discussed and had a dialogue about the group dynamics and how the following discussions could be developed based on the summary.

The moderators´ intention was that all group members should participate in the discussion in an equal way. This intention was challenged, however, as some of the participants, though hints about this, took more space than others. This may mean that some experiences and reflections with relevance for the results did not appear.

The discussions lasted for 50–70 min, were digitally recorded, and gave rich and meaningful content results. The Arabic moderator transcribed the discussions in Arabic directly into Swedish as she had qualified translation skills. An external translator with extensive experience of moderating focus groups and transcribing data, qualified translation skills and an appropriate academic degree was used for the Somali discussions. To minimize the risk of interpreter bias, uniform guides for translation and transcription were used and 10% of each transcript was checked by a third bilingual person. In addition, the moderators were asked to give feedback on the final results as to the extent to which they thought they agreed with their own view of what the participants had told them. A more detailed presentation of the transcription process is also accounted for elsewhere [24].

One of the three moderators carried out discussions as well as transcribed and translated the transcripts in order to reduce the risk of misinterpretation or loss of information. However, this procedure was not possible in the Somali speaking focus groups, which is a limitation. The analysis was conducted in Swedish, and the quotes were translated into English in the results section to make them available to non-Swedish readers.

### Analysis

The analysis was based on Graneheim and Lundman’s method for content analysis [33]. The analysis was carried out in several steps, mainly by the first author. However, to secure the connection between empirical data and categories, all researchers took part in the last steps of the analysis. Reflexivity was considered in all parts of the analysis process in the form of acknowledging preconceptions as a factor that could potentially influence the process and the results during the analysis.

The goal of the content analysis in this study was to achieve categories representing visible and obvious components of experiences and needs, referred to as an expression of the manifest content [33, 34]. In concrete terms, this means that the results consist of descriptions of the content of the discussions, i.e. there is a low degree of interpretation of the data, as well as of abstraction, in the analysis process [34].

The transcripts were read several times to gain an overall understanding of the content. This was followed by distinguishing meaning units according to the aim of the study, i.e. the experiences and needs concerning the content and distribution of health-related information for newly arrived asylum seekers in Sweden. The meaning units were then condensed into descriptions close to what was communicated in the discussions and a categorization of the condensed material. This was done by a sorting based on similarities and differences in the content and resulted in categories of experiences and needs related to health information, which were labeled according to their content. In concrete terms, this means that similar opinions and experiences made up the base for each category. The divergent opinions and experiences that were expressed gave rise to four categories dissimilar to each other. As all the empirical data clearly belonged to at least one of the categories, the data were judged to be theoretically saturated [32].

Despite the low degree of interpretation in the manifest analysis process, the next step was to check the consistency between the categories and their content and the empirical data (the transcribed and translated discussions). The last step in the process was to formulate the descriptive content of the categories [34].

## Results

Four qualitatively different categories emerged from the experiences and needs related to health information that were expressed in the focus group discussions. Based on the manifest content, they were termed: ‘concrete instructions and explanations’, ‘contextual knowledge’, ‘a variation of sources’ and ‘enabling communication’. Significant citations are provided to illustrate the content of the categories and to ensure an empirical foundation.

### Concrete instructions and explanations

Some of the health-related information that the refugees had received during their time in the new country was experienced as important and useful on a daily basis. The information included concrete advice from health professionals on how to deal with symptoms or/and health problems, when actions are needed, how to act, how to prevent sickness, where to seek help, and ways to proceed in the health care system when necessary. Information about results of tests, screening and other forms of examinations was also experienced as valuable, regardless of whether the results confirmed a state of good health or reported detection of an illness or infection.

*– I did what the doctor said and I got better immediately. The information I got really helped me.*

Explanations about symptoms, specific diseases, processes related to illness or disease, and differences between treatment and examination options were judged to be very important. However, the extent of the information was expressed as being insufficient in terms of being regarded as tools to manage health issues. A need was expressed for concrete guidelines about procedures related to the acquisition and management of medication, and instructions as to how to behave when they had preferences concerning an interpreter or the gender of the professional.

*– What if it’s a male doctor? How can I show myself naked, dear God? That’s haram (forbidden).*

The communication of health information was sometimes experienced as leading to confusion, as it could differ from person to person, from one institution to another and from information retrieved via Internet compared to that received from health professionals. In addition, the content of the information could be contradictive to valid knowledge in the home country. How to achieve reliable health information was expressed as a need; this meant where to find it and information about how to estimate the reliability of information from different sources.

*– But there is a problem...I asked him how to treat it and he said with medication, but then I said there was another doctor in xx (a community) who said that it needed to be operated. But then the first one said that he didn’t recommend me to operate it. And now I don’t know what to do. I have a time booked, should I have the operation or not? One doctor says that it needed to be operated and the other in XX (a community) says that it is better not to do it since it will come back though it is operated.*

### Contextual knowledge

Information about health, health problems and the health care system based on a contextual perspective was highlighted as a need to decrease the risk of misunderstandings and unnecessary suffering. This area of information included/should include specific health risks and diseases in Sweden, as well as the common opinion of valid examinations related to them and instructions on how to protect to them.

*– … and I know that every society has diseases that are specific to them that are widespread or are endemic there. We don’t know what these are in Sweden, no one has told us … about what kinds of diseases you can get in Sweden and that Swedes get … and how they protect themselves from them.*

There was also a desire for knowledge about the health care system in Sweden, how it is organized, legislated and regulated, rights and charges related to the use of health care, and specific routines related to the provision of health care. Other forms of contextual knowledge that were expressed to be needed included information about beliefs or norms about the provision and practice of health care, why some examinations, tests and cures are not regarded as relevant in Sweden, and the reasons for a restricted provision of medicines.

*– You want to be able to understand how things work here, such as health care, the school system, everything. For example, if you get an appointment with the doctor, a specific time for each person and if you don’t come to the doctor at the appointed time you have to pay a certain fee for not having come. So you have to keep times and you have to understand what the doctor says to you, then everything can go well.*

Knowledge about the Swedish perspective of sexuality and the female body was also described as desirable, in order to gain an impression of how such subjects are approached for adolescents in compulsory schools. It was also judged to be important to be informed about individual actions that should be taken to fulfill societal expectations of one’s responsibility for one’s own health.

*– Last year they gathered young people in the school and informed them about where girls who had started to menstruate could get hold of contraceptives. They give the young people information about things like this, but the parents should be given information about important things. Yes, we parents first and then they can tell our children.*

### A variation of sources

Written health information was experienced as preferable to verbal because of the possibility that it could be supported with translation. The written information received and/or retrieved was in the form of notices to health examinations, results of tests, folders about specific conditions and general health issues. However, situations were described in which verbal advice or information that had been received had been valuable or even crucial for the outcome. Information in oral form had been given by relatives, friends, other refugees, staff in pharmacies, migration office servants, and health care professionals and at the course in civic orientation. An intentional search for health information on web pages was also described.

One experience was that health information is not available and applicable to everyone because of analphabetism, deficient language skills or lack of knowledge of where to find it. More options for presentations of health information were requested and desired. It should not only be available in the health care context, but also at schools, group activities, courses and seminars at refugee camps, asylum houses and mosques, as well as on the radio, TV, YouTube, web pages and in public halls. Both formal and informal channels are expressed as important.

*– I live a little bit outside of town so the ambulance needs at least 10 min to get there, and my husband sometimes goes into a diabetes coma. So we asked if they could give us information, come to school and give us the bases of first aid if a child faints, falls, cuts himself, things like that, so that we know what we should do instead of sitting and screaming in the telephone or doing something wrong. My husband who has diabetes might die if I give him more insulin … we asked them about this but then they said that it was difficult. I was surprised, why is it difficult? A printed paper can’t be so difficult?*

*– They could also distribute special folders with the most important web sites for health, for example health centers and addresses. If we have a question about health or what we should do in acute cases. They should give us things like that in written form.*

### Enabling communication

Based on good and/or bad experiences, suggestions were given to facilitate the health information exchange and to enable communication to mutual satisfaction. Professionals that exerted themselves to understand or be understood were appreciated. The experience was that health professionals most often did not consider potential problems caused by language difficulties when planning health care visits or other forms of contact. Messages given in Swedish on an answering machine were described as meaningless. It was also experienced as problematic to contact health care by phone due to automatic answers in combination with button choices.

*– I can for example wake up and find that my son is sick. Then I want to call the health center. The one who answers is a taped voice. We get surprised here. We’re so new … the only thing I hear is*” *press button, press, press!” But this is very hard for us. Finally I give up. This is very hard for us. It would be so good if they found a solution to this problem so that we know how we should communicate. How we should make an appointment. Or that I have to go there directly.*

Situations with efficient information exchange were described to include ones in which professionals are informed about the specific needs of newly arrived individuals, with their experiences of fleeing and having insufficient language skills and a foreign background. To avoid misunderstandings, the experience was that information and explanations should be mediated by a person that is familiar with the conditions in the country of origin.

*– Somalis who are doctors or nurses should be either on TV or the radio or that they organize a seminar, so that you can get knowledge and information from someone who also comes from your country.*

More time should be spent on health care to enable the information change, and information should be repeated so that it can be understood and judged as adequate.

*– I mean that the information should be repeated, maybe at the hospital, that they (the health care professionals) build up the person’s confidence, that they shouldn’t do it just one time.*

## Discussion

The refugees’ experience that information received in the form of concrete instructions and explanations is important, valuable and useful indicates a concordance between the refugees’ information needs and the adequacy of the information given. As the individuals in the focus groups were heterogenic in terms of country of origin, age, sex and education, the results indicate that the information given to them by health care professionals meet individuals’ personal needs. This is in line with the idea of a health literate health care organization (HLHO) [35], whose intention is to compensate for individuals’ limited health literacy to increase the abilities of vulnerable persons to navigate in the health care system, to facilitate access to community-based health literacy resources, and to be responsive to individual needs and improve health outcomes [35–37].

However, another part of the results, showing that health professionals were experienced as often neglecting potential problems stemming from language difficulties, is contradictory to that conclusion. According to Swedish law, health care professionals have a duty to inform patients and to involve them as a part of their health care and to secure that the information that is given is understood [38]. The use of cultural mediators is a possible way to improve the communication between health care and refugees [39–41]. Cultural mediators are usually people from the same culture and/or country as the refugees, but with experiences and knowledge about the new country including culture, norms and structures. Thus, they can give information and discuss issues from the refugees’ perspective, and answer questions in the refugees’ native language. Further, they can sustain a dialogue about the information, comparing situations and procedures in the old and the new country [42].

The need experienced by the refugees to be informed with valid and consequent information, including explanations about differences between treatment and examination options, makes clear a desire on the part of the refugees to make their own well-founded decisions concerning health issues and to actively participate in their contacts with health care. This desire indicates a self-image of a capable person with his or her own responsibility to take care of up-coming health issues. Realizing this self-image in a new country places demands on the health organization to spread valid and appropriate information and to provide empowering interventions [43]. Bravo et al. [43] have described that health literacy, personal capacity, self-efficacy, sense of meaning and coherence about health problems, perceived control and feelings of being respected by their health care providers are indicators of patient empowerment [43]. According to The United Nations 2030 Agenda for Sustainable Development [44], vulnerable persons must be empowered, and some of the goals of the agenda clearly relate to health literacy by emphasizing lifelong learning opportunities, healthy lives and the promotion of well-being [44]. Inadequate individual and organizational health literacy acts as a barrier to personal engagement and empowerment [45]. Thus, the importance of concrete instructions and explanations is clear.

The experienced need of more contextual information about the health care system, health care regulations, health issues, health behavior et cetera in the new country confirms the conception of health literacy as a context-dependent phenomenon [1], meaning that it is possible for the same individual to be health literate in one context or society but not in another [46]. In the most of today’s Western world societies, there are expectations (sometimes unspoken) on the individual to have sufficient knowledge about rights and obligations, to make decisions with regard to health issues, and to take one’s own initiatives in order to maintain and improve health, to make own decisions, to judge when support is needed and to know where to turn when help is needed. In all, this means that being active, independent and self-determined constitutes the “patient’s role”. Arriving from a country where the role is being passive or patient and in which the decision is made from above may mean a move on the health literacy continuum from a sufficient level to an inadequate level of health literacy [1].

The World Health Organization (WHO) [47] has stated that the main force for the health in a population is situated outside the health care systems, and thus health literacy is a responsibility for the society as a whole, i.e. not only for health care organizations. Even if this was not focused on or mentioned in the discussions, everyday life in general includes a great number of more or less conscious decisions that in one way or another may influence health. Some of these may be context related, and thus important to be aware of when a person comes to a new country. To fulfill the expressed need of more contextual health information, this could be made a basic topic in civic orientation courses for newly arrived refugees.

The expressed need of a variation in health information sources, formal as well as informal, also calls for the provision of information outside the health care system. The benefits of oral information that were described, retrieved at sources that are less or more formal, illustrate the importance of the communicative dimension of health literacy [48–50]. The examples given by the refugees about spreading health information through group activities, courses and seminars at refugee camps, asylum houses and mosques, as well as on the radio, TV, YouTube, web pages and in public halls, seem creative and worth making use of. Still, the most important aspect for the health care system as well as for other information providers is that health information must be available to everyone, i.e. even to persons who are analphabets or have deficient language skills. Individualized, person-centered and targeted information is desirable [46] to secure that information reaches the persons for whom it is intended.

The category concerning the enablement of communication also illustrates the importance of a person-centered approach in the health care situation, including both the past and present of the person and his/her context. Giving messages (in Swedish) on answering machines or giving automatic responses (also in Swedish) with instructions for button choices when a person calls for help seems unreflected from the perspective of a refugee. The suggestions given by the refugees, including taking into account potential language difficulties in all forms of communication, that more time should be reserved for visits and that professionals should be informed about the specific needs of newly arrived individuals, with their experiences of fleeing and having a foreign background, might serve as guidelines in the development of the health examination for asylum seekers and other forms of health care.

An important attribute of an HLHO is that it includes the population served in the design, implementation and evaluation of health information and services [35], and the results of our study show that the experiences of refugees are worth listening to on an organizational level when planning health promoting or health care activities for them. Health literacy on an organizational level is described to increase individuals’ perception of their self-efficacy in dealing with health-related issues and, as a consequence of that, increases their motivation to take care of themselves [51]. One way to reduce existing information inequality in patients at a health care center could be to train healthcare workers in health literacy to raise awareness and knowledge about which patients might need extra attention and consideration and how to assess the information need. Furthermore, the 10 item questionnaire (HLHO-10) [35] could be used to identify and improve specific areas within the health care organization when adopting to the HLHO perspective.

The experiences and needs concerning health-related information that was described by the refugees are related in different ways to the HLS-EU model of health literacy [2]. All of the categories included expressions for both needing and wishing to be health literate by having the abilities to access, understand, appraise and apply health related information, which all are central aspects of the model. The refugees gave examples of ways for society to strengthen their abilities to cope with their health issues. This is also in line with the model that suggests that health literacy may be influenced not only by the individual but also by social, societal and environmental factors. According to the model, such actions may have a positive impact on health care use, costs for health care, health behaviors, health effects, empowerment and equity [2].

### Methodological considerations

This was an explorative study aimed at gaining and spreading knowledge about the refugees‘experiences, described in their own words and based on their own views. The limited selection of refugees (originating from four countries only) may mean that the applicability of the results to other groups of refugees is limited. Another limitation with respect to the applicability of the results is the recruitment of participants from courses in SFI and Civic Orientation. This selection excluded young people (below 18 years) and elderly people (older than 65) as well as people with poor health to the degree that they are not able to participate in those courses.

Collecting data in the refugees’ native languages means obtaining comprehensive information from a group that is otherwise often excluded from research. However, the process of translating data from the languages of origin to Swedish contributes to limitations concerning reasonableness and the accuracy of the results, as the expressions from each participant underwent an interpretation process, which may misrepresent the participants’ actual expressions. These limitations may be further reinforced in the presentation of the results in English.

The pre-understanding of the authors may also have influenced the research process. However, this background may be judged as a resource in capturing the different experiences that were sought in this study. Such experiences may have contributed to the appropriateness of the results of the analysis as well as to the credibility. Another factor that contributes to credibility was that all researchers took part in the last step of the analysis process, which included a check of the consistency between the categories and their content and the empirical data. The fact that the discussions gave rich and meaningful content indicates that the selection of participants was appropriate. According to Graneheim & Lundman [33, 34], this also contributes to credibility. A way to further strengthen the credibility of the results would have been to ask some of the participating refugees to give feedback on the findings. This was not done.

In qualitative research, the intent is to gain theoretical saturation, which may be achieved by the emergence of patterns during the analysis [31]. The rule of thumb in focus group research is to create three to four groups and then decide whether further groups are needed [27]. The situation in the data collection, with some participants taking more space than others, may mean that some experiences were not expressed. Further, the use of focus groups may have prevented some participants from speaking freely. Opinions other than those described in the four categories may thus exist.

## Conclusions

The refugees experienced that concrete instructions and explanations were valuable and applicable. However, necessary health information is not always accessible or adapted according to their requirements. Further, the refugees’ language and their prior knowledge were experienced as not always being considered in meetings with health care professionals. Additional information about health issues and the health care system was desired from a variety of sources. Based on these experiences, health literacy among refugees may be improved if the following points are employed by health professionals:

- Adapt the information to the target group by using the native language and channels that are available to them, and ensure that the information can be understood.
- Do not assume that refugees possess similar knowledge or view of health care/health as natives.
- Include information that the target group considers important in addition to the information you want to disseminate.

## Supplementary information

**Additional file 1.** Discussion guide: Description of the questions that supported the moderator in the focus group discussions.

## Data Availability

The datasets used and/or analyzed during the current study are available from the corresponding author on reasonable request.

## Abbreviations

HEA: Health Examination for Asylum seekers
HLHO: Health literate health care organization
SFI: Swedish for migrants
WHO: World Health Organization
