# Supplementary material for: Experiences and needs concerning health related information for newly arrived refugees in Sweden
Source: BMC Public Health. 2020 Jul 1;20:1044. doi: 10.1186/s12889-020-09163-w (PMC7331281; doi:10.1186/s12889-020-09163-w)
Supplement: Supplementary file 1 — Additional file 1. Discussion guide: Description of the questions that supported the moderator in the focus group discussions. [file 12889_2020_9163_MOESM1_ESM.docx]

**Discussion guide**

**Introduction**

You are invited to this focus group session to discuss the accessibility, understanding, estimation and feasibility of health-related information. You have the common experience of being a refugee and having taken part in a health examination for asylum seekers (HEA) or a civic orientation course. However, the upcoming discussion focuses on your experiences of health-related information in general, i.e. not necessary connected to the HEA och the civic orientation course.

**Questions to discuss**

What do you do to get information about something that has to do with your health?

How do you do this, where do you look for information?

What information is easy/difficult to get?

What would make it easier to get the information?

With regard to the information about health and health questions that you establish contact with

How easy/difficult is it to understand health-related information in general?

What makes the information easy/difficult to understand?

Can you give examples of when the contents of the information have been easy/difficult to understand?

How important is the situation in which the information is given?

What is the importance of the source of the information for understanding the meaning of the information?

What is the importance of the situation in which the information is given for understanding the meaning of the information?

How can it be made easier for newly arrived refugees to understand information that has to do with health?

What do you do to judge whether the health information you receive or procure is correct and dependable?

Can you give examples of when it has been easy/difficult to judge whether the information has been correct and dependable?

What are your thoughts when you judge whether the information is correct and dependable?

How can it be made easier for newly arrived refugees to judge whether health information is correct and dependable?

How useful is the health information you receive or procure?

Can you give examples of when you have used the health information you have received or procured in a practical situation?

In what way was the information useful?

Can you give examples of when the health information that you have received or procured not been possible to use in a practical situation?

What was the reason why the information could not be used?

How can it be made easier for newly arrived refugees to use health information in a practical situation?
